# Lupin poisoning: a review

**DOI:** 10.3389/ftox.2025.1547535

**Published:** 2025-04-09

**Authors:** Meye Bloothooft, Pien Cremers, Sükriye Güven, Stijn J. Stoutjesdijk, Mara Jiron, Mark Wessel, Marcel A. G. Van Der Heyden

**Affiliations:** ^1^ Department of Medical Physiology, Division of Heart and Lungs, University Medical Center Utrecht, Utrecht, Netherlands; ^2^ Honours Program CRU^+^ Bachelor, University Medical Center Utrecht, Utrecht, Netherlands

**Keywords:** lupin, alkaloid, *L. albus*, *L. mutabilis*, anticholinergic syndrome, ACS, poisoning, intoxication

## Abstract

**Introduction:**

Lupin beans are the seeds of plants from the Fabaceae family. These beans are rich in protein and used for human consumption for several millennia. Their popularity is still increasing. Some species produce beans with high alkaloid contents, specifically of spartaine and lupanine. Without proper processing, consuming these beans can result in lupin poisoning that causes anticholinergic syndrome. We systematically analyzed all case reports describing lupin poisoning in humans in order to define most observed clinical findings, treatment options and outcome.

**Methods:**

We screened Pubmed and Google scholar for human case reports on lupin poisoning. Obtained full-text papers in any language were screened for eligibility. Demographics, time-to-symptoms, treatment and outcome were analyzed. Symptoms were categorized using the ICD11 classification.

**Results:**

Twenty-seven case reports describing 33 patients were obtained. Poisoning occurred in all age groups and sexes equally. Most frequent symptoms were bilateral mydriasis (n = 25), xerostomia (n = 25), blurred vision (n = 17), lightheadedness (n = 14), weakness (n = 11). Onset of symptoms was typically within 60 min. In most cases no treatment was required, and symptoms resolved within 24 h after which patients were discharged. Two of four children in the cohort required ICU admission and one died.

**Discussion:**

Lupin poisoning is rare and requires most often a conservative clinical approach. However, in children the effects are more severe than in adults. Frequency of lupin poisoning may rise due to increased popularity of the beans as an alternative protein source.

## 1 Introduction

Legumes, like lupin beans, are nutritious as well as affordable and are increasingly sold and consumed in today’s society. They are regarded as an addition to, or alternative for soybean protein, and are rich in protein and fibers. Also, they may offer positive effects on blood pressure, blood lipid levels and a person’s weight (Pereira et al., 2022). The consumption of lupin beans has increased over the last decades and is expected to increase even further. Since 2000 the production of lupin has increased from approximately 90,000 ton per year to 450,000 ton per year in Europe (Pereira et al., 2022; Lucas et al., 2015). Although the consumption of lupin beans has positive health benefits, it is less known that unprocessed lupin beans can have negative side effects by inhibiting the parasympathetic nervous system (Resta et al., 2008).

**FIGURE 1:**
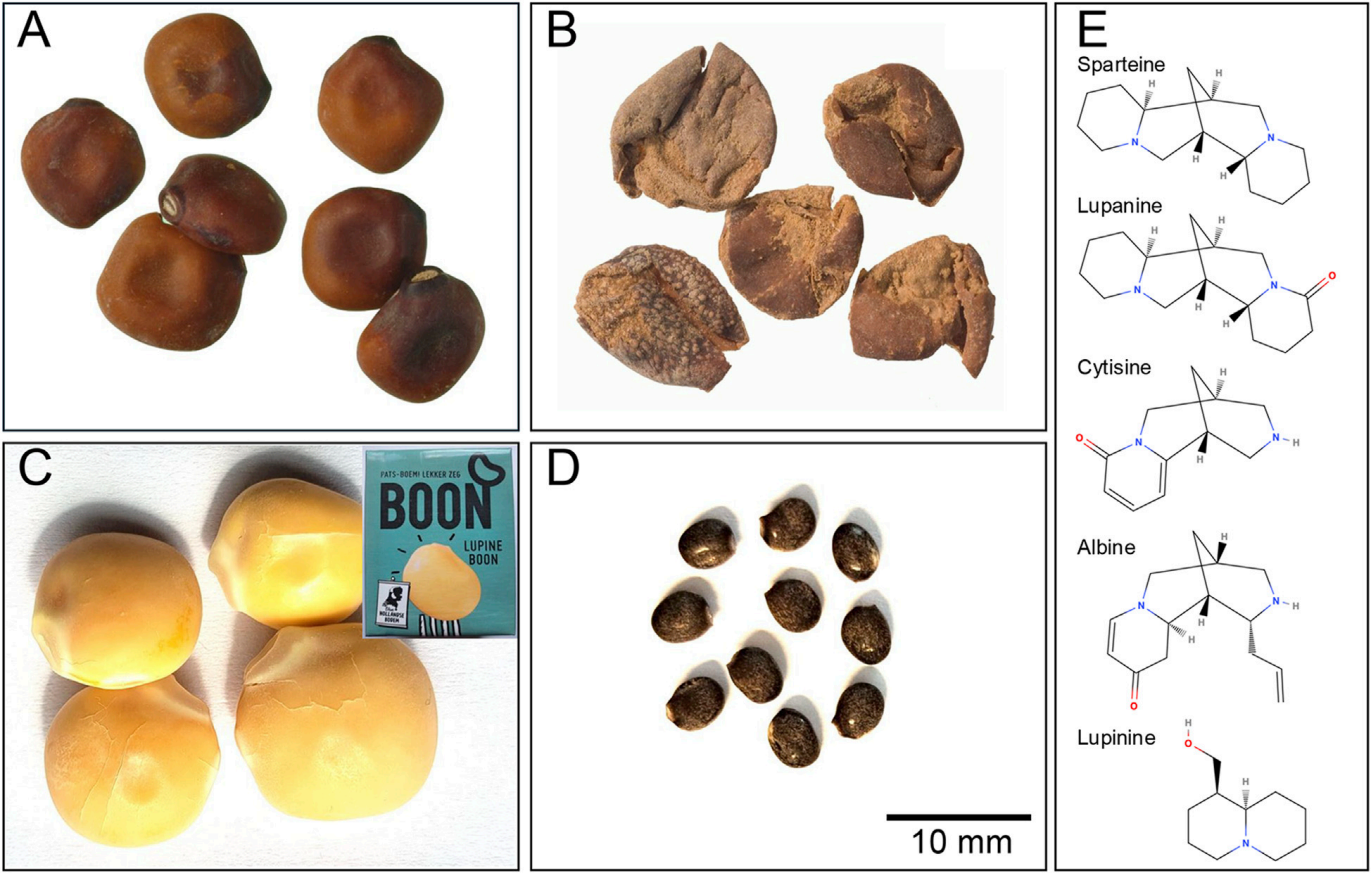
**(A)** Desiccated seeds and **(B)** discarded skins of white lupin or termis bean (*Lupinus alba*), from the Roman period (first to early second century AD) recovered from domestic refuse deposits at Quseir al-Qadim, Egypt (Van der Veen, 2011), Plate 13. Copyright M. Van der Veen, photos J. Morales (reproduced with approval of the original authors)). **(C)** Processed 21st century lupin seeds from the supermarket (inset container). **(D)** lupin seeds from the horticultural popular Washington lupin (*Lupinus polyphyllus*). Scale bar for panels **(A–D)**. **(E)** Chemical structures of alkaloids present in lupin seeds (drawn using MolView).

The genus of Lupins belongs to the family of Fabaceae, which comprises in total of approximately 280 species, of which 13 originate from the Old World (i.e., Europe and North Africa) (Eckstein et al., 2023). Domesticated lupin beans include four major species, namely, *Lupinus albus*, *Lupinus angustifolius*, *Lupinus luteus*, and *Lupinus mutabilis*. Two species, *L. albus* and *L. mutabilis*, have been used for consumption for many era (Figure 1) (Carvajal-Larenas et al., 2016). *L. albus* originates from the European Balkan region and is widespread in the Mediterranean region (Huyghe, 1997). *L. mutabilis* (or “Andean lupin,” also known as Tarwi) originates from the South American Andean highlands, where it was domesticated around 650 BC (Atchison et al., 2016). Archeological finds from the same region also strongly suggest the use of wild or other domesticated species in the sixth millennium BC (Atchison et al., 2016). Egyptian and Greek records indicate the use of lupin beans as a human food source in ancient times, as far back as 2000 BC [e.g., Zhukovsky (Zhukovsky, 1929); Gladstones (Gladstones, 1970)]. Furthermore, the medicinal use of lupin beans in various forms, including its so-called debittering water, was already apparent in ancient times as, for example, described by Pliny (AD 23/24 – AD 79) in his *Naturalis Historia* and shortly by Galenus (AD 129–199) in his *De alimentorum facultatibus* (Kurlovich, 2024).

Unprocessed lupin beans contain alkaloids, which are toxic and therefore unsuitable for consumption. These alkaloids bind to and block nicotinic and muscarinic cholinergic receptors, thereby (partially) inhibiting the parasympathetic nervous system. This is known as anticholinergic syndrome. Furthermore, the alkaloids sparteine and lupanine inhibit Na^+^ and K^+^ ion channels. *L. mutabilis* possesses high amounts of alkaloids, around 30 g/kg, with lupanine being the most prominent (±50%) alkaloid*. L. albus* contains around 10 g/kg alkaloids, with lupanine as the most prominent alkaloid (±70%). Other alkaloids present in lupin beans are, for example,: sparteine, lupinine, albine and cytisine (Figure 1E) (Schrenk et al., 2019).

Lupin beans that are available in supermarkets are processed and directly edible. To make unprocessed lupin beans suitable for consumption they need to be stripped of alkaloids by washing in a time demanding debittering process (Lowen et al., 1995). The beans should be soaked in a large amount of water, so-called debittering water, and cooked for 10 min each day for at least 5 days in a row before the beans are detoxified and safe for consumption, as described in a case report (Smith, 1987). The observed parasympathetic inhibition in patients after consumption of unwashed or insufficiently washed lupin beans or debittering water is known as lupin poisoning or intoxication (Ozkaya et al., 2021; Luque Marquez et al., 1991).

Since there is a shortfall in knowledge on the negative health outcomes of incorrectly processed lupin beans, we analyzed all existing case reports on lupin bean poisoning in humans, for demographics, symptoms and treatment. This review aims to increase awareness amongst healthcare workers and the general public of the symptoms, diagnosis and treatment of lupin bean poisoning.

## 2 Materials and methods

### 2.1 Case report collection

A retrospective analysis of existing scientific literature on lupin poisoning was done. The review was conducted according to the PRISMA (Preferred Reporting Items for Systematic Reviews and Meta-Analysis) guidelines. Relevant papers were found *via* PubMed and Google Scholar using advanced search techniques with the following search terms: lupin AND toxicity, lupin AND poisoning, lupin toxicity OR lupin poisoning, and lupin bean intoxication. Articles in all languages (i.e., English, Spanish, Italian, French, German, Swedish, Hebrew), were assessed that came up with these search terms. Additional case reports were identified based on the reference lists of the articles found during the search in PubMed and Google Scholar. Since some case reports were written in Spanish, additional case reports were searched for in Spanish in Google, with search terms: lupinus intoxicacion or altramuz intoxicacion. Papers written in other languages than English were translated via Google Translate and subsequently reviewed by native speakers with a medical background.

**FIGURE 2:**
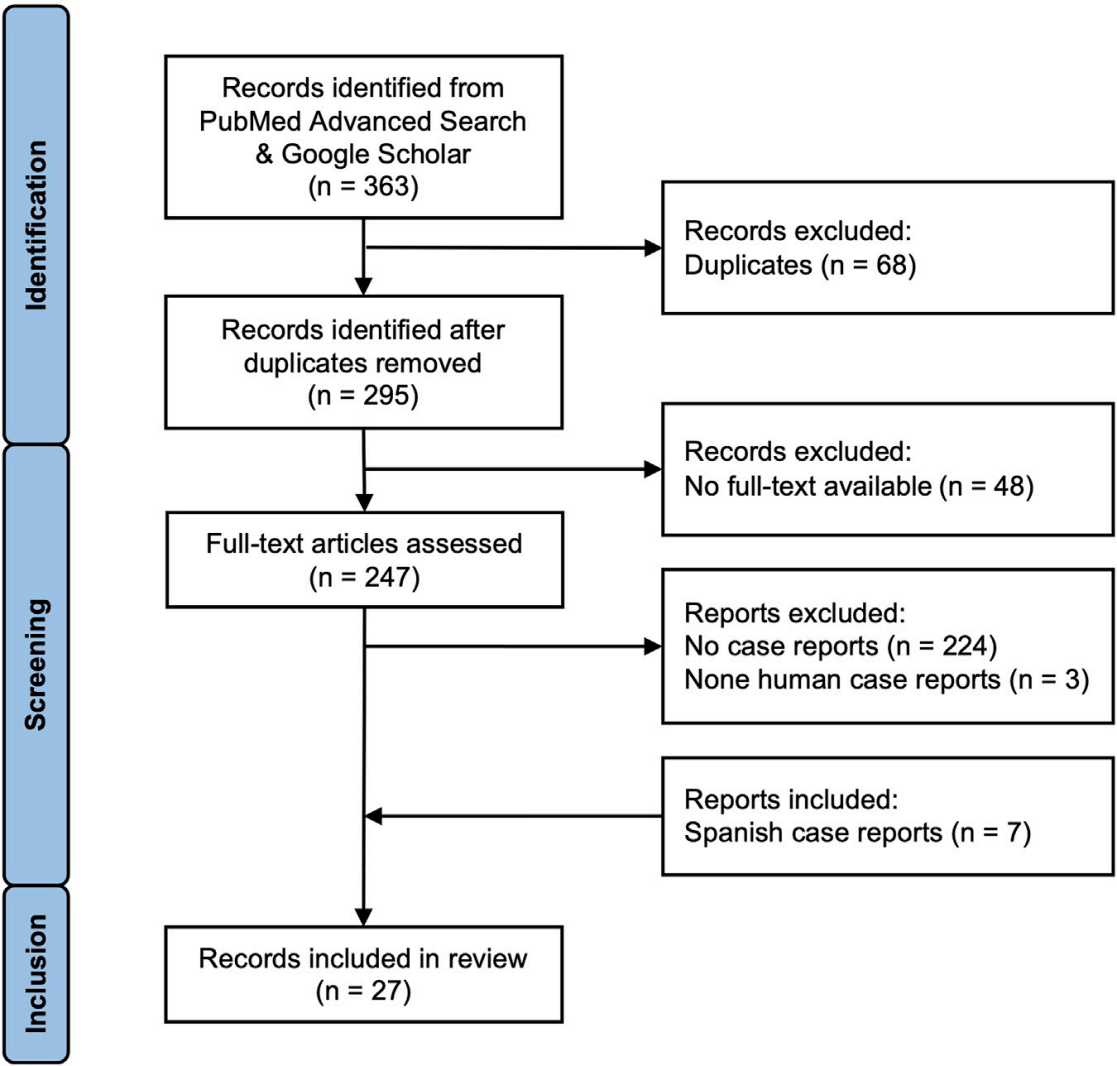
Flowchart of the in- and exclusion criteria of lupin poisoning case reports.

The identified articles were screened and checked for inclusion (Figure 2). Papers were included in our analysis, if they were/had: human case-reports, accessible in full-text, no lupin poisoning with other diagnoses, cases with no medical history of other diseases that can cause anticholinergic symptoms, symptom description and background information, and cases without medication known to produce analogous symptoms. This resulted in 27 case reports that were used for further analysis.

### 2.2 Data extraction

To minimize interobserver variability, all case reports in this study were reviewed and interpreted by a minimum of two authors of the current paper. The following parameters, if available, were extracted from the case reports: patient characteristics; clinical symptoms; timeframe of the onset; symptoms; treatment; recovery time; type, dose and species of lupin.

### 2.3 Data analysis

We used the International Classification of Diseases (ICD) 11, the diagnostic classification system of the World Health Organization (WHO), to accurately categorize the symptoms of lupin poisoning. For analysis some data were categorized (e.g., age).

## 3 Results

**FIGURE 3:**
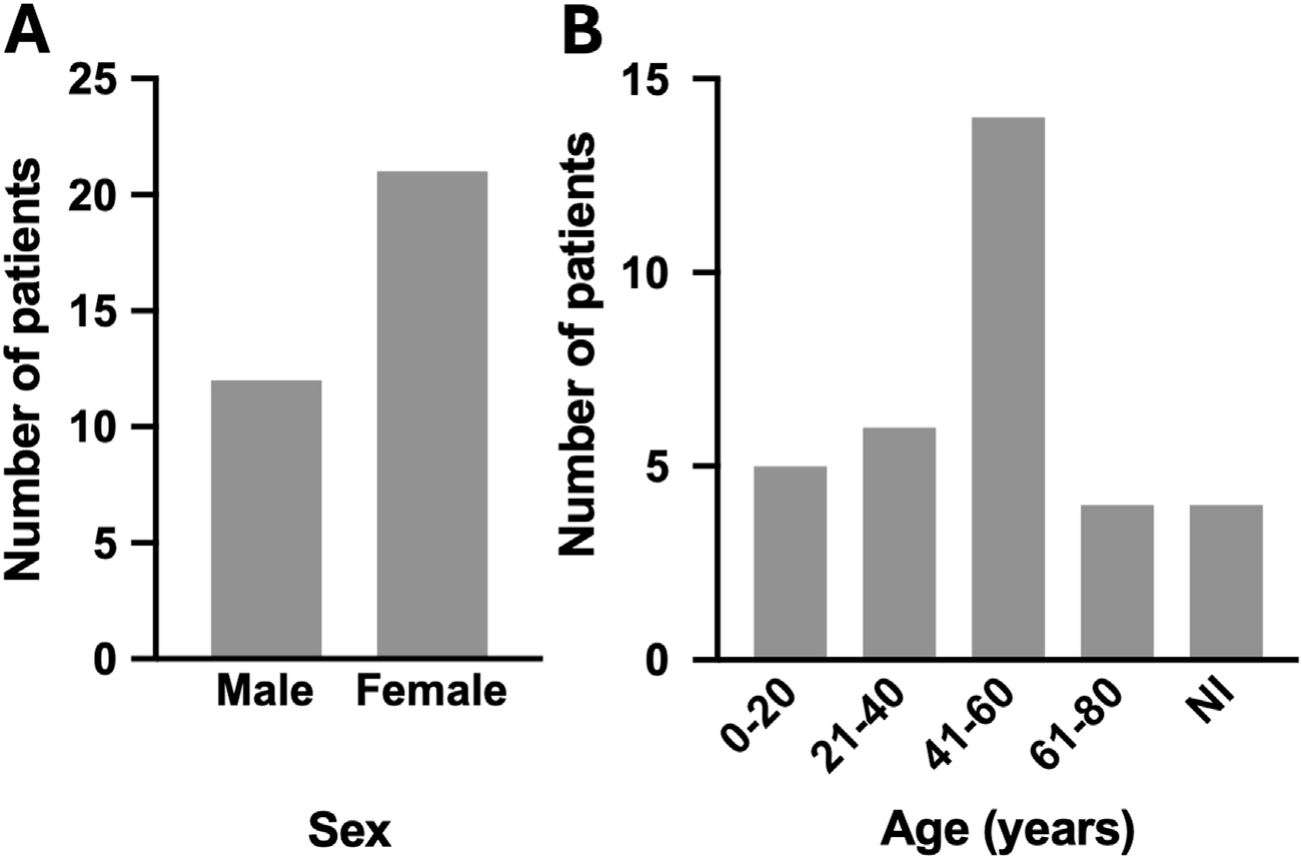
Sex **(A)** and age **(B)** distribution of lupin poisoning patients. NI = not included in the case report.

The search for lupin poisoning case reports resulted in 27 reports (Ozkaya et al., 2021; Pingault et al., 2009; Ortega Duarte et al., 2013; Luque Marquez et al., 1991; Malmgren et al., 2016; Litkey and Dailey, 2007; Li et al., 2017; Jamali, 2011; Gapany et al., 1983; Flores-Pamo et al., 2018; Di Grande et al., 2004; Daverio et al., 2014; Awada et al., 2011; Agid et al., 1988; Tsiodras et al., 1999; Smith, 1987; Schmidlin-Mészáros, 1973; Petraroia, 1926; Lowen et al., 1995; Lahoud et al., 2021; Alessandro et al., 2017; Camacho Saavedra and Uribe Uribe, 1995; Carazo Barrios and De la Fuente Cañete, 2022; Cidad et al., 2013; Lorente Esparza et al., 2021; Moreno González and Del Caño Garrido, 2021; Vivancos Gallego and Machín, 2014) describing 33 patients between 1926 and 2022 (Table 1). Patients that presented with lupin poisoning were of all ages (1–73 years old, Figure 3B) and occurred in both males and females, however slightly more females were poisoned (female n = 21, males n = 12, Figure 3A). Relatively often a female around 30–50 years old was described (n = 10). In some case reports patients stated that they had taken the beans/debittering water for health purposes: “because of healing properties,” “a suggestion of a friend against high cholesterol,” or “as diet, because of the diuretic effect” (Camacho Saavedra and Uribe Uribe, 1995; Cidad et al., 2013; Gapany et al., 1983; Ortega Duarte et al., 2013; Moreno González and Del Caño Garrido, 2021; Alessandro et al., 2017).

**TABLE 1:** Characteristics of the lupin poisoning cases.

Amount/type of ingested lupin	Time before onset	Treatment	Recovery time	Remarks	Sex	Age (years)	First author	year of publication	Origin of paper	Language of article
3 gr lupin beans each month for 8 years (*L. Albus*)	-	Benzhexol	20 months	Remained with symptoms	Female	28	Agid	1988	France	English
Homeopathic medication (*L. mutabilis*)	6 h	IV fluids	12 h		Male	48	Alessandro	2017	Argentina	English
Yellow lupin bean	-	None	Within 24 h		Male	37	Awada	2011	France	French
∼200–300 gr lupin beans	2 h	-	-		Female	34	Awada	2011	France	French
250 mL debittering water (*L. mutabilis*)	1 h	-	16 h		Female	40	Camacho	1995	Peru	Spanish
150 mL debittering water (*L. mutabilis*)	1 h	None	-		Male	43	Camacho	1995	Peru	Spanish
2 L enema (*L. mutabilis*)	30 min	-	48 h		Female	48	Camacho	1995	Peru	Spanish
Lupin flour (*L. albus*)	30 min	Analgetics, IV fluids	8 h		Female	41	Carazo	2022	Spain	Spanish
Debittering water (*L. mutabilis*)	4 h	-	24 h		Female	59	Cidad	2013	Spain	Spanish
10 lupin beans	1 h	-	Within 24 h		Female	6	Daverio	2014	Italy	English
Lupin beans	some hours	None	Within 12 h		Female	51	Di Grande	2004	Italy	English
Lupin beans (*L. mutabilis*)	-	IV fluids, ceftriaxone and mannitol	Within 72 h	ICU admission	Male	1	Flores-Pamo	2018	Peru	English
250 mL debittering water	30 min	-	Within 12 h		Female	50	Gapany	1983	Israel	Hebrew
Lupin beans	4 h	Urinary catheterization	-		Male	44	Jamali	2011	Australia	English
Lupin beans	hours	None	Within 12 h		Female	50	Lahoud	2021	Lebanon	English
Lupin beans	hours	-	-		Male	-	Lahoud	2021	Lebanon	English
300 mL debittering water	15 min	Urinary catheterization	Within 24 h		Male	63	Li	2017	United States	English
100 mL debittering water	-	-	Within 24 h		Female	-	Li	2017	United States	English
Lupin beans (*L. albus*)	30 min	None	Within 12 h		Female	46	Litkey	2006	United States	English
Debittering water (*L. mutabilis*)	30 min	IV fluids, anti-emetics, activated charcoal	24 h		Female	52	Lorente	2021	Spain	Spanish
Two handfuls of lupin beans	14 h	IV fluids, urinary catheterization	Within 12 h		Female	35	Lowen	1995	Australia	English
80–100 gr lupin beans (*L. albus*)	-	IV fluids	Within 4 days		Male	50+	Malmgren	2016	Sweden	Swedish
500 mL debittering water	1 h	-	Within 48 h		Male	19	Marquez	1991	Spain	English
Soup with lupin beans (*L. mutabilis*)	15 min	IV fluids	24 h		Female	57	Moreno	2021	Spain	Spanish
500 mL debittering water (*L. mutabilis*)	30 min	IV fluids, nasogastric tube, urinary catheterization	Within 48 h		Female	38	Ortega Duarte	2013	Peru	Spanish
Two handfuls of lupin beans	30 min	Midazolam and fentanyl, orogastric lavage	Within 5 days	ICU admission	Male	12	Ozkaya	2021	Türkiye	English
Lupin beans	hours	Anti-emetic, hypertensive drugs, stimulating drugs	-	Died	Male	1	Petraroia	1926	Italy	Italian
2 scones with lupin flour (*L. albus*)	1 h	-	Within several hours		Female	73	Pingault	2009	Australia	English
Pancakes with lupin flour (*L. albus*)	15 min	-	Within 12 h		Female	66	Pingault	2009	Australia	English
Lupin beans	-	Gastric lavage	Within 24 h		Male	-	Schmidlin	1973	Switzerland	German
Lupin beans	-	-	-		Female	-	Smith	1987	Canada	English
Debittering water	-	None	Within 24 h		Female	72	Tsriodoras	1999	United States	English
Debittering water (*L. mutabilis*)	hours	IV fluids	-		Female	45	Vivancos	2014	Spain	Spanish

**FIGURE 4:**
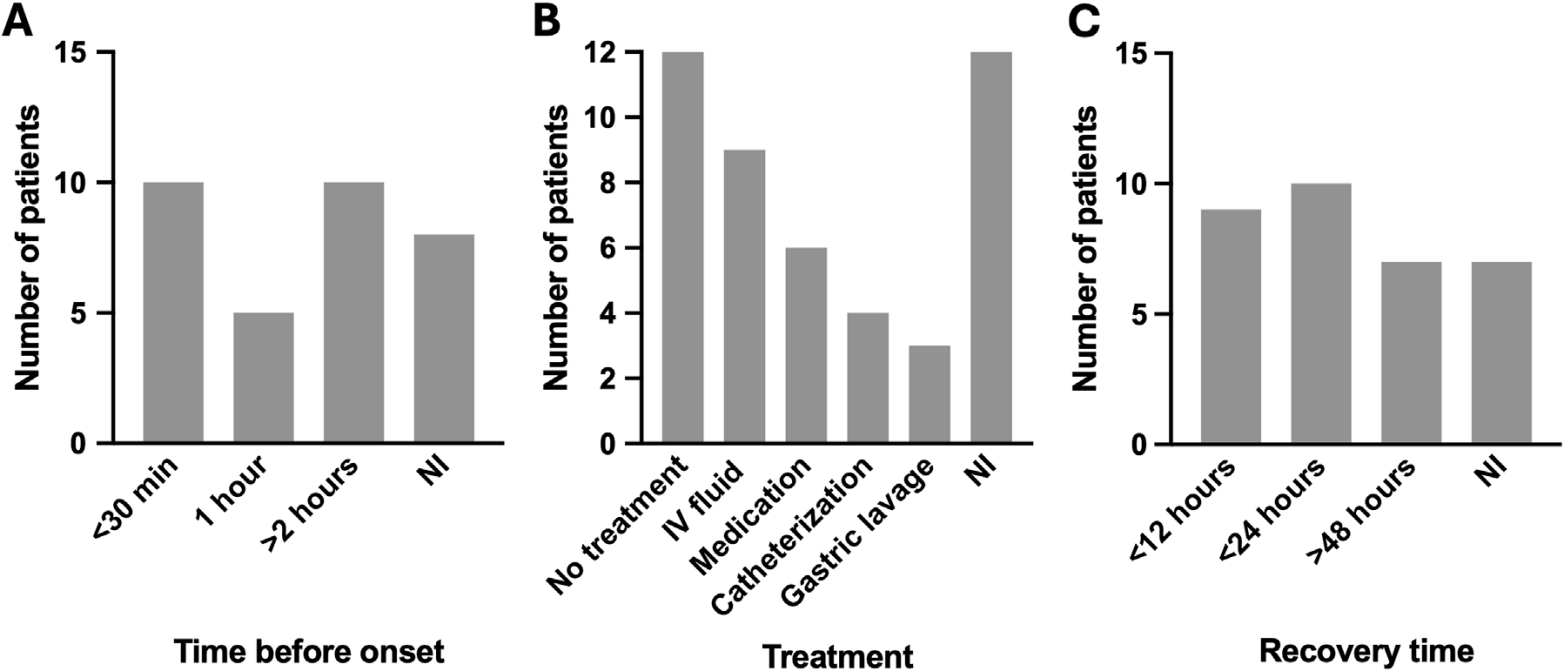
Time before onset symptoms **(A)**, treatment **(B)** and recovery time **(C)** of lupin poisoning patients. NI = not included in the case report.

45% of the patients presented the first signs of poisoning within an hour after intake of lupin (n = 15). Although time to onset of symptoms varied between 15 min and 14 h (Figure 4A). Most patients ingested beans or debittering water, but also cases of lupin containing flour or homeopathics were mentioned, (Table 1). There was no clear correlation between type of beans [*L. albus* (n = 6) vs. *L. mutabilis* (n = 10)] or type of intake [beans (n = 17) vs. debittering water (n = 11)] and the severity of the symptoms. Since the dose of ingested beans is not described in most of the papers, it is hard to find causality between amount of beans/water ingested and the outcome.

Sixty-nine different symptoms were described in the 33 different patients (Table 2 shows an overview of all symptoms reported n > 1; Supplementary Appendix 1 shows all symptoms per case), of which many occurred only once (n = 25). All symptoms (n = 262) were classified via the ICD11 system. Symptoms were mostly seen in the digestive system (n = 69), visual system (n = 63), nervous system (n = 29) and circulatory system (n = 23). Most common symptoms were mydriasis (n = 25), xerostomia (n = 25), blurred vision (n = 17), lightheadedness (n = 14) and weakness (n = 11). Symptoms seem to be progressing over time when patients present in the clinic quickly after the poisoning. For example, symptoms worsen or new symptoms appear.

**TABLE 2:** Clinical symptoms associated with lupin poisoning. Only symptoms with n ≥ 2 are included in the table. All clinical symptoms per case are included in Supplementary Appendix 1. Symptoms are classified according to the ICD11 system.

System (number of symptoms)	Symptoms	Frequency
Digestive (69)	Xerostomia	25
	Nausea	10
	Distended abdomen	8
	Abdominal pain	6
	Emesis	5
	Decreased bowel sound	4
	Dysphagia	3
	Erythematous pharynx	2
	Tympanic to percussion	2
Visual (63)	Mydriasis	25
	Blurred vision	17
	Minimally/unreactive to light	8
	Photophobia	4
	Difficulty focussing	3
	Dry eyes	3
Nervous (29)	Lightheadedness	14
	Headache	3
	Babinski reflex	2
	(Hemi)dystonia	2
Circulatory (23)	Tachycardia	10
	Hypotensive	5
	Palpitations	4
	Hypertensive	2
General (22)	Weakness	11
	Malaise	6
	Lethargy	2
	Restlessness	2
Mental and Behavioral (20)	Anxiety	6
	Confusion	5
	Irritable	4
	Altered mental status	2
Skin (17)	Dry skin	4
	Flushing	3
	Warm skin	3
	Diaphoresis	2
	Pallor	2
Urinary (10)	Urinary retention	8
	Distended bladder	2
Respiratory (5)	Tachypnea	3

58% of the patients recovered within 24 h (n = 19) after ingestion of lupin (Figure 4C), resulting in discharge from the hospital. Within these 24 h, most patients remained under observation but did not need any medical intervention. If any treatment was given, it was against one of the symptoms, like (urinary) catheterization (n = 4) for urinary retention and IV fluids (n = 9) to keep vitals stable (Table 1; Figure 4B). Gastric lavage or activated charcoal was mentioned a few times as an option to be used to prevent the alkaloids from absorbing. Gastric lavage was used twice and active charcoal once.

Two out of the four children described in the case reports ended up in the intensive care unit (Flores-Pamo et al., 2018; Ozkaya et al., 2021), but also recovered after a few days and were discharged eventually (Daverio et al., 2014) (Table 1). One of the children, a 1-year-old boy, died (Petraroia, 1926). All but one patient recovered completely. This case presented with an intake of lupin beans over the time course of 8 years (Agid et al., 1988). She gradually recovered in the 20 months after quitting eating the beans. She remained with some complaints after that. Given the relatively low intake compared to the other cases and the long time frame before the onset of symptoms, there might have been confounding etiologies. However, such were not described in the case report and symptoms disappeared after ending the intake of lupin beans.

## 4 Discussion

Lupin beans are consumed more and more in today’s society. They are considered healthy and a substitute for soybeans (Pereira et al., 2022). However, when beans are not correctly stripped of the toxic alkaloids the beans can be toxic or even lethal (Lowen et al., 1995; Schmidlin-Mészáros, 1973; Petraroia, 1926). The high amount of alkaloids in the beans affect the parasympathetic nervous system and cause anticholinergic syndrome (Ozkaya et al., 2021; Luque Marquez et al., 1991). In this review the human case reports on lupin bean poisoning have been collected and data regarding the poisoning, symptoms and treatment were extracted.

The beans that were consumed that led to poisoning were *L. albus* and *L. mutabilis*. The poisoning symptoms generally occurred within 1 h after consumption of the beans, or the debittering water of the processing of the beans. Most common symptoms of poisoning were with dryness (mouth, eyes, skin), sight problems, abdominal problems, muscle (tension, contractions) and urinary retention. In most cases a wait and see policy was sufficient and patients recovered mostly within 24 h and were discharged. When treated, some patients got gastric lavage or activated charcoal as treatment to remove the beans from the stomach. To maintain stable vitals, some patients received IV fluids. In case of urinary retention patients received catheterization. Extra care should be taken with children, since a few cases described ICU admission or lethal outcome (Flores-Pamo et al., 2018; Ozkaya et al., 2021; Petraroia, 1926; Daverio et al., 2014).

In other cases, which we were not able to access the full-text paper, also pediatric death was described. The citation of these cases by Schmidlin-Mészáros (1973) describes a 10-year-old child that died after 3 h, with similar symptoms as described in our cases, e.g., mydriasis and convulsions. And a 1.5-year-old child that also died after eating 5–10 g of lupin beans. So, of all the reported poisoning in children (n = 6) three died, two were admitted to the ICU and one had toxication symptoms, but recovered on its own (Ozkaya et al., 2021; Flores-Pamo et al., 2018; Petraroia, 1926; Daverio et al., 2014; Schmidlin-Mészáros, 1973). While for all the adult cases reports, this has not occurred. Only one other case cited by Schmidlin-Mészáros (1973) describes an adult that got into a coma, but recovered. Obviously, case reports are only written on the out of the ordinary cases and possible confounding etiologies are not always described and some cases are from almost a century ago. But it is striking that children are more severely affected after poisoning than adults. Therefore, it might potentially be beneficial to treat children with gastric lavage soon after consumption to reduce the amount of alkaloids absorbed in the body and worsen the poisoning. Also, monitoring of vital signs is important, since for most cases the symptoms worsen over time.

Most alkaloids have a half-life within 12 h in humans, which aligns with the recovery of patients within 12–24 h in general. The alkaloids are partially metabolized via the liver and CYP2D6 (Schrenk et al., 2019). This enzyme is known to have polymorphisms, which can lead to a different rate in metabolism of the alkaloids. This might explain why some patients take more time to recover than others. Alkaloid concentrations between 10–50 mg/kg are estimated to cause poisoning, which might be even lower for children. It is not known how frequent lupin poisoning occurs, but probably not often, given that most consumed lupin beans are from the supermarket, and those are already debittered (Schrenk et al., 2019).

Besides lupin poisoning, lupin allergy also exists. An allergic reaction is not caused by alkaloids, but by conglutin proteins that result mainly in anaphylaxis, asthmatic or urticaria. Cross reactivity with peanut allergy is often seen. Lupin allergy is expected to affect a few percent of the population, although studies on lupin allergies are limited (Sanz et al., 2010; Villa et al., 2020). So, lupin allergy has a different clinical representation, and therefore also different treatment compared to poisoning.

A narrative review like this gives much insight in lupin poisoning by comparing the different case reports. However, we rely on the available information in the case reports. The reports often provide an elaborate description of the cases, but sometimes the description is limited. For example, the amount or type of lupin beans is not always described but would give valuable insight in lupin poisoning. So, the largest limitation in this study remains the availability of data.

Although lupin poisoning prevalence is low and outcomes are usually good, proper treatment can avoid symptoms going from bad to worse, definitely in children. With this review we aimed to describe the symptoms and treatment of lupin poisoning by extracting data of existing lupin poisoning case reports. Therewith we hope that healthcare professionals can diagnose and treat those patients as best as possible. And since it is expected more lupin will be consumed it is important to increase awareness and knowledge on lupin intoxication.

## Data Availability

The original contributions presented in the study are included in the article/Supplementary Material, further inquiries can be directed to the corresponding author.
