# Supplementary material for: Lupin poisoning: a review
Source: Front Toxicol. 2025 Apr 9;7:1547535. doi: 10.3389/ftox.2025.1547535 (PMC12014556; doi:10.3389/ftox.2025.1547535)
Supplement: Supplementary file 1 [file Table1.docx]

Supplementary Material

**Appendix 1.** Symptom classification per case.

| **paper** | **symptom** | **symptom category** | **Classification (ICD11)** |
| --- | --- | --- | --- |
| Agid | weakness of limbs | weakness | general system |
|  | hemidystonia | (hemi)dystonia | nervous system |
|  | amyotrophy | amyotrophy | nervous system |
|  | fasciculations | fasciculations | nervous system |
|  | dysphagia | dysphagia | digestive system |
|  | dysarthria | dysarthria | speech system |
|  | difficulty writing and knitting | difficulty writing and knitting |  |
|  | dystonic | dystonia | nervous system |
|  | diffuse hyperflexia | hyperflexia | nervous system |
|  | extensor plantar responses | babinski relfex | nervous system |
|  | progressive weakness | weakness | general system |
| Alessandro | blurred vision | blurred vision | visual system |
|  | dizziness | light headedness | nervous system |
|  | palpitations | palpitations | circulatory system |
|  | mydriasis | mydriasis | visual system |
| Awada (case 1) | general malaise | general malaise | general system |
|  | palpitations | palpitations | circulatory system |
|  | blurred vision | blurred vision | visual system |
|  | light headedness | light headedness | nervous system |
|  | dry mouth/tongue | xerostomia | digestive system |
|  | sinus tachycardia | sinus tachycardia | circulatory system |
|  | bilateral mydriasis | mydriasis | visual system |
|  | minimally reactive to light | minimally/unreactive to light | visual system |
| (case 2) | malaise | general malaise | general system |
|  | palpitations | palpitations | circulatory system |
|  | light headedness | light headedness | nervous system |
|  | blurred vision | blurred vision | visual system |
|  | dilated pupils | mydriasis | visual system |
|  | dryness of mouth | xerostomia | digestive system |
| Camacho (case 1) | dizziness | light headedness | nervous system |
|  | headache | headache | nervous system |
|  | dry mouth | xerostomia | digestive system |
|  | difficulty focussing | difficulty focussing of the eyes | visual system |
|  | photophobia | photophobia | visual system |
|  | emesis | emesis | digestive system |
|  | hypotension | hypotensive | circulatory system |
|  | agitated | irritable | mental or behavioural system |
|  | dry skin | dry skin | skin system |
|  | mydriasis | mydriasis | visual system |
| (case 2) | blurred vision | blurred vision | visual system |
|  | dry mouth | xerostomia | digestive system |
|  | restlessness | restlessness | general system |
|  | warm skin | warm skin | skin system |
|  | mydriasis | mydriasis | visual system |
| case (3) | nausea | nausea | digestive system |
|  | emesis | emesis | digestive system |
|  | abdominal pain | abdominal pain | digestive system |
|  | distended abdomen | distended abdomen | digestive system |
|  | high breathing frequency | tachypnea | respiratory system |
|  | dry mouth | xerostomia | digestive system |
|  | pale | pallor | skin system |
|  | decreased bowel sounds | decreased bowel sounds | digestive system |
| Carazo | mydriasis | mydriasis | visual system |
|  | general malaise | general malaise | general system |
|  | dizziness | light headedness | nervous system |
|  | nausea | nausea | digestive system |
|  | abdominal pain | abdominal pain | digestive system |
|  | dry mouth | xerostomia | digestive system |
|  | hypotension | hypotensive | circulatory system |
|  | abdominal distension | distended abdomen | digestive system |
| Cidad | confusion | confusion | mental or behavioural system |
|  | agitated | irritable | mental or behavioural system |
|  | blurred vision | blurred vision | visual system |
|  | mydriasis | mydriasis | visual system |
| Daverio | headache | headache | nervous system |
|  | blurry vision | blurred vision | visual system |
|  | photophobia | photophobia | visual system |
|  | nausea | nausea | digestive system |
|  | tachycardia | sinus tachycardia | circulatory system |
|  | dry oral mucous | xerostomia | digestive system |
|  | bilateral mydriasis | mydriasis | visual system |
|  | unreactive to light | minimally/unreactive to light | visual system |
| Di Grande | weakness | weakness | general system |
|  | anxiety | anxiety | mental or behavioural system |
|  | dry mouth | xerostomia | digestive system |
|  | bilateral mydriasis | mydriasis | visual system |
|  | lid drop | ptosis | visual system |
| Flores | perioral cyanosis | perioral cyanosis | skin system |
|  | severe cough | severe cough | respiratory system |
|  | stridor | stridor | respiratory system |
|  | irritable | irritable | mental or behavioural system |
|  | altered conciousness | altered conciousness | mental or behavioural system |
|  | shallow breathing | shallow breathing | respiratory system |
|  | dilated sluggishly reactive pupils | mydriasis | visual system |
|  | dry mucous membrane | xerostomia | digestive system |
|  | distended abdomen | distended abdomen | digestive system |
|  | tympanic to percussion | tympanic to percussion | digestive system |
|  | altered mental status | altered mental status | mental or behavioural system |
| Gapany | severe headache | headache | nervous system |
|  | dizziness | light headedness | nervous system |
|  | nausea | nausea | digestive system |
|  | blurred vision | blurred vision | visual system |
|  | dry mouth/dry oral mucosa | xerostomia | digestive system |
|  | weakness | weakness | general system |
|  | tachycardia | sinus tachycardia | circulatory system |
|  | wide pupils | mydriasis | visual system |
|  | unresponsive to light | minimally/unreactive to light | visual system |
|  | difficulty urinating | urinary retention | urinary system |
|  | swelling of the bladder | distended bladder | urinary system |
| Jamali | dizziness | light headedness | nervous system |
|  | blurred vision | blurred vision | visual system |
|  | dry mouth/mucous membranes | xerostomia | digestive system |
|  | dilated pupils | mydriasis | visual system |
|  | non reactive pupils | minimally/unreactive to light | visual system |
|  | unable to pass urine | urinary retention | urinary system |
|  | warm skin | warm skin | skin system |
|  | dry skin | dry skin | skin system |
| Lahoud (case 1) | bilateral mydriasis | mydriasis | visual system |
|  | dryness of mouth | xerostomia | digestive system |
|  | dry eyes | dry eyes | visual system |
|  | anxious | anxiety | mental or behavioural system |
|  | weak light reflexes | minimally/unreactive to light | visual system |
|  | abnormal commodation-convergence reflex | abnormal commodation-convergence reflex | visual system |
| case 2 | stomach pain | abdominal pain | digestive system |
|  | anxious | anxiety | mental or behavioural system |
|  | dry mouth | xerostomia | digestive system |
|  | dry eyes | dry eyes | visual system |
|  | general malaise | general malaise | general system |
| Li | emesis episodes | emesis | digestive system |
|  | generalized weakness | weakness | general system |
|  | difficulty urinating | urinary retention | urinary system |
|  | confusion | confusion | mental or behavioural system |
|  | visual hallucination | visual hallucination | mental or behavioural system |
|  | distended abdomen | distended abdomen | digestive system |
|  | quiet bowel sounds | decreased bowel sounds | digestive system |
|  | dry skin | dry skin | skin system |
|  | dilated pupils | mydriasis | visual system |
| case 2 | light headedness | light headedness | nervous system |
|  | nausea | nausea | digestive system |
|  | generalized weakness | weakness | general system |
|  | vision change | blurred vision | visual system |
|  | anxiety | anxiety | mental or behavioural system |
|  | decreased bowel movements | decreased bowel movements | digestive system |
| Litkey | dry mouth | xerostomia | digestive system |
|  | blurry vision | blurred vision | visual system |
|  | facial flushing | flushing | skin system |
|  | confusion/ difficulty completing a sentence and staying focused | confusion | mental or behavioural system |
|  | not able to look far | difficulty focussing of the eyes | visual system |
|  | hot to touch | warm skin | skin system |
|  | irritable | irritable | mental or behavioural system |
|  | tachycardia | sinus tachycardia | circulatory system |
|  | hypertensive | hypertensive | circulatory system |
|  | bilateral mydriasis | mydriasis | visual system |
|  | unreactive to light | minimally/unreactive to light | visual system |
|  | soft, non tender abdomen | soft abdomen | digestive system |
|  | decreased bowel sounds | decreased bowel sounds | digestive system |
| Lorente | general malaise | general malaise | general system |
|  | dizziness | light headedness | nervous system |
|  | photophobia | photophobia | visual system |
|  | blurred vision | blurred vision | visual system |
|  | xerostomia | xerostomia | digestive system |
|  | dry eyes | dry eyes | visual system |
|  | dysphagia | dysphagia | digestive system |
|  | nausea | nausea | digestive system |
|  | mydriasis | mydriasis | visual system |
|  | erythematous pharynx | erythematous pharynx | digestive system |
| Lowen | dry mouth | xerostomia | digestive system |
|  | blurred vision | blurred vision | visual system |
|  | difficulty swallowing | dysphagia | digestive system |
|  | muscular weakness | weakness | general system |
|  | exhaustion | exhaustion | general system |
|  | urinary urgency/retention | urinary retention | urinary system |
|  | feelings of impending doom | anxiety | mental or behavioural system |
|  | grossly dilated but reactive pupils | mydriasis | visual system |
|  | mild sinus tachycardia | sinus tachycardia | circulatory system |
|  | distended bladder | distended bladder | urinary system |
| Malmgren | unconcious | unconcious | mental or behavioural system |
|  | dizziness | light headedness | nervous system |
|  | nausea | nausea | digestive system |
|  | bilateral mydriasis | mydriasis | visual system |
|  | low blood pressure | hypotensive | circulatory system |
|  | abdominal pain | abdominal pain | digestive system |
|  | distended abdomen | distended abdomen | digestive system |
|  | confused/no clear thinking | confusion | mental or behavioural system |
|  | dry mouth | xerostomia | digestive system |
|  | raspy voice | raspy voice | speech system |
|  | urine retention | urinary retention | urinary system |
| Marquez | generalized weakness | weakness | general system |
|  | impaired short range sight | difficulty focussing of the eyes | visual system |
|  | dry mouth | xerostomia | digestive system |
|  | palpitations | palpitations | circulatory system |
|  | urine retention | urinary retention | urinary system |
|  | hyporeactive bilateral mydriasis | mydriasis | visual system |
|  | tachycardia | sinus tachycardia | circulatory system |
|  | abundant ventricular extrasystoles | extrasystoles | circulatory system |
|  | trigeminism | trigeminism | circulatory system |
| Moreno | dry mucous membrane | xerostomia | digestive system |
|  | dizziness | light headedness | nervous system |
|  | blurred vision | blurred vision | visual system |
|  | paresthesia of the upper limbs | parasthesia in the upper limbs | skin system |
|  | xerostomia | xerostomia | digestive system |
|  | mydriasis | mydriasis | visual system |
| Ortega Duarte | sensation of instability | sensation of instability |  |
|  | blurred vision | blurred vision | visual system |
|  | dry skin | dry skin | skin system |
|  | dry mouth | xerostomia | digestive system |
|  | anxiety | anxiety | mental or behavioural system |
|  | generalized weakness | weakness | general system |
|  | hyoptension | hypotensive | circulatory system |
|  | areactive mydriasis | mydriasis | visual system |
|  | abdominal pain | abdominal pain | digestive system |
|  | distended abdomen | distended abdomen | digestive system |
|  | nausea | nausea | digestive system |
|  | urine retention | urinary retention | urinary system |
|  | tympanic to percussion | tympanic to percussion | digestive system |
| Ozkaya | generalized tonic clonic seizure muscles | generalized tonic clonic seizures | nervous system |
|  | vomiting | emesis | digestive system |
|  | hypertensive | hypertensive | circulatory system |
|  | tachycardia | sinus tachycardia | circulatory system |
|  | high breathing frequency | tachypnea | respiratory system |
|  | bilateral mydriasis | mydriasis | visual system |
|  | weak light reflexes | minimally/unreactive to light | visual system |
|  | increased muscular tone | hypertonia | nervous system |
|  | dry oral mucosa | xerostomia | digestive system |
|  | flushing | flushing | skin system |
|  | piloerection | piloerection | skin system |
|  | babinsky reflex | babinski reflex | nervous system |
| Petraroia | pale | pallor | skin system |
|  | closed eyes | Lagophthalmos | visual system |
|  | hypotonia | hypotonia | nervous system |
|  | unresponsive | unresponsive | nervous system |
|  | distended abdomen | distended abdomen | digestive system |
|  | gagging | gagging | digestive system |
|  | abdominal paralysis | abdominal paralysis | digestive system |
|  | altered mental status | altered mental status | mental or behavioural system |
|  | hypotension | hypotensive | circulatory system |
|  | convulsions | convulsions | nervous system |
| Pingault (case 1) | lethargy | lethargy | general system |
|  | difficulty mobilizing | difficulty mobilizing |  |
|  | dry mouth | xerostomia | digestive system |
| (case 2) | dry mouth | xerostomia | digestive system |
|  | blurred vision | blurred vision | visual system |
|  | lethargy | lethargy | general system |
|  | light headedness | light headedness | nervous system |
|  | sinus tachycardia | sinus tachycardia | circulatory system |
|  | mildly dilated pupils | mydriasis | visual system |
|  | urine retention | urinary retention | urinary system |
| Schmidlin | weakness | weakness | general system |
|  | diaphoresis | diaphoresis | skin system |
|  | stomach/bowel pain | abdominal pain | digestive system |
|  | mydriasis | mydriasis | visual system |
| Smith | uncoordinated | uncoordinated |  |
|  | dizzy | light headedness | nervous system |
|  | nausea | nausea | digestive system |
| Tsiodras | vomiting | emesis | digestive system |
|  | nausea | nausea | digestive system |
|  | diaphoresis | diaphoresis | skin system |
|  | blurred vision | blurred vision | visual system |
|  | generalized weakness | weakness | general system |
|  | tachycardia | sinus tachycardia | circulatory system |
|  | tachypnea | tachypnea | respiratory system |
|  | dilated pupils | mydriasis | visual system |
|  | minimally reactive to light | minimally/unreactive to light | visual system |
| Vivancos | blurred vision | blurred vision | visual system |
|  | general malaise | general malaise | general system |
|  | dizziness | light headedness | nervous system |
|  | dry mouth | xerostomia | digestive system |
|  | photophobia | photophobia | visual system |
|  | tachycardia | sinus tachycardia | circulatory system |
|  | confusion | confusion | mental or behavioural system |
|  | restlessness | restlessness | general system |
|  | flushing | flushing | skin system |
|  | distended abdomen | distended abdomen | digestive system |
|  | decreased bowel sounds | decreased bowel sounds | digestive system |
|  | erythematous pharynx | erythematous pharynx | digestive system |
